# Cervical Cancer Screening: Analyzing Recent Guideline Changes Against Current Trends of Human Papillomavirus (HPV) Risk Factors

**DOI:** 10.7759/cureus.117745

**Published:** 2026-10-10

**Authors:** Susan E Garber, Leselle Pierre-Romain

**Affiliations:** 1 Public Health: Preventive Medicine, St. George's University School of Graduate Studies, True Blue, GRD

**Keywords:** cervical cancer risk factors, cervical cancer screening, epidemiologic trends, human papillomavirus (hpv), preventive medicine, screening guidelines

## Abstract

Cervical cancer screening is an important aspect of public health and preventive medicine and contributes to reducing cervical cancer-related deaths. Cervical cancer screening guidelines have changed over time, shifting toward less frequent screening recommendations and an increased age of screening onset. These changes have been influenced by advances in screening technology, cost-effectiveness considerations, and the widespread implementation of Human papillomavirus (HPV) vaccination. Epidemiologic trends demonstrate increasing HPV-related risk factors, declining screening adherence, and barriers related to knowledge, access, and patient-provider communication. This narrative review examines cervical cancer screening guideline changes in relation to current epidemiologic trends in HPV risk factors. Existing literature was reviewed to evaluate changes in screening recommendations, patterns of screening participation and adherence, shifts in HPV-related risk behaviors, and patient-provider communication barriers influencing screening uptake. Although updated screening guidelines are supported by technological advancements and cost-effectiveness considerations, increasing trends in HPV risk factors and reduced screening adherence may limit the effectiveness of less frequent screening strategies. This narrative review analyzes cervical cancer screening guideline changes in relation to current epidemiologic trends in HPV risk factors, an important consideration when evaluating screening policy decisions.

## Introduction and background

Cervical cancer screening is an important aspect of public health, which helps prevent cervical cancer-related deaths. Worldwide, cervical cancer is the fourth leading cause of cancer incidence and mortality among women [1]. Routine screening and early treatment can prevent 69.7% of cervical cancer deaths [2]. Screening methods include the Papanicolaou (Pap) test, also known as the Pap smear, the human papillomavirus (HPV) test, or an HPV/Pap co-test [3]. HPV is a virus that can cause cervical cancer and is transmitted via sexual contact. Worldwide, HPV is the most common sexually transmitted disease [4]. Many individuals infected with HPV naturally resolve the infection, while others have persistent infection, which increases the risk of cervical cancer. Recent guideline changes have recommended a decreased testing frequency, with screening at a later age due to natural resolution of the virus in most patients, technical advancements, cost-effectiveness, and the accessibility of HPV vaccination [3,5]. However, epidemiologic trends show an increase in patients overdue for screening from 14% in 2005 to 23% in 2019, worsening with the COVID-19 pandemic in 2020 [6]. Trends show a younger age of initial sexual intercourse, an increasing number of sexual partners, lack of protective measures, increased nicotine use, and limited knowledge regarding HPV and cervical screening, with these risk factor trends increasing among younger generations [1,6-10]. The current epidemiological trends of HPV risk factors imply an increased risk of HPV infection, therefore increasing cervical cancer risk. However, current guidelines recommend a decreased frequency of testing. Through this narrative review, recent changes to cervical cancer screening guidelines are analyzed in relation to current HPV risk factor trends. The variables examined are screening guideline evolution, screening participation and adherence, HPV-related risk behavior trends, and patient-provider communication barriers; a consideration for screening policies.

## Review

Methods

Study Design

A narrative review was chosen as the study design to synthesize evidence on recent cervical screening guideline changes and analyze them against current HPV risk factor trends. Choosing a narrative review allowed for comprehensive integration of epidemiological and clinical data, as well as literature on screening policies.

Search Strategy and Data Sources

The review process was conducted from May through October 2025. Databases such as PubMed, Scopus, Google Scholar, WHO, CDC, and NCI were used. Search terms included: “cervical cancer screening guidelines”, “HPV risk factors”, “cervical cancer screening frequency”, “sexual behavior trends”, “cervical cancer screening adherence”, “Pap test”, “HPV vaccination impact”. Boolean operations, such as “cervical cancer screening AND HPV trends”, were used to enhance the impact of the searching methods. Sources used focused on years 2000-2025 to capture the evolution of the cervical cancer guideline changes as well as post-HPV vaccination initiation. All sources were in English.

Eligibility Criteria

Inclusion criteria included peer-reviewed sources, epidemiological significance, cost-effectiveness analysis, HPV risk factor significance, information on cervical cancer screening guidelines, and systematic reviews/meta-analyses. Exclusion criteria included editorial papers, conference abstracts, case reports, or studies that were not directly significant, defined as studies that did not explicitly address cervical cancer screening practices, HPV infection or prevention, or population-level health outcomes related to screening access and adherence.

Selection Process and Data Extraction

Titles were first analyzed for relevance and significance, where significance was defined as the study’s clear contribution to understanding cervical cancer screening guidelines, HPV risk factors, or epidemiologic trends. If a title was deemed significant, the article’s abstract was then reviewed for eligibility. If the abstract met the inclusion criteria, the full text of the article was examined to extract data and evidence for inclusion in the narrative review. A total of 21 peer-reviewed articles were included for synthesis in this narrative review. A single reviewer conducted the literature search, article selection, and data extraction.

Synthesis of Findings

The findings were analyzed and organized into four themes: guideline evolution/cost-effectiveness analysis [3,5,11-14], screening participation/adherence trends [6,15,16], HPV risk factors/epidemiologic trends [1,2,4,8-10,17,18], and finally communication, comprehension, and provider/patient barriers [7,19-21]. Articles within each theme were analyzed based on their relevance to screening recommendations, population-level adherence, epidemiologic risk factors, and behavioral or systemic barriers influencing the implementation of cervical cancer screening practices. Cervical cancer screening was considered according to the screening methods and guidelines applicable to the time period of each study. One figure and one table were compiled to summarize, clearly present, and better visualize the guideline comparisons and epidemiologic risk factor trends.

Thematic analysis of findings

Guideline evolution/cost-effectiveness analysis

Cervical cancer screening guidelines have evolved substantially over time, particularly with the introduction of HPV testing as an alternative to traditional cytology-based screening. Before 2012, annual Pap testing was commonly practiced. In 2012, the United States Preventive Services Task Force (USPSTF) shifted away from annual screening in an effort to reduce potential harms associated with over-screening, including unnecessary diagnostic procedures and anxiety resulting from false-positive findings. Screening recommendations changed to every 3 years for individuals aged 21-29 and every 5 years with Pap/HPV co-testing for those aged 30-65, with screening generally discontinued after age 65 in appropriately screened individuals [11]. These changes represented an important transition from frequent cytology-based screening toward longer screening intervals.

In 2020, the American Cancer Society (ACS) further revised its guidance, recommending that screening begin at age 25 and favoring primary HPV testing every five years [3]. Cost-effectiveness analyses have supported the transition toward HPV-based screening and management strategies [5]. Pilot studies implementing these guidelines supported HPV testing as both a safe and effective approach for extending screening intervals to five years [12,13]. These findings contributed to the shift toward HPV-based screening and longer intervals between cervical cancer screening tests. As screening intervals become longer, patient adherence remains important because individuals must still return for screening according to the recommended schedule.

In 2024, the World Health Organization (WHO) endorsed HPV testing as the global gold standard, recommending screening every 5-10 years and projecting equivalent mortality reduction compared with 3-year Pap testing [14]. This progression reflects a global shift toward less frequent screening, later screening initiation, and greater reliance on HPV testing. While less frequent screening may reduce unnecessary testing and provide benefits related to healthcare resources and cost-effectiveness, patients who already struggle with follow-up may become further delayed under extended screening schedules. Therefore, although evidence supports longer screening intervals with HPV-based testing, patient adherence remains an important consideration when implementing these recommendations.

Screening participation/adherence trends 

Although cervical cancer screening guidelines recommend specific time intervals, patients are not always adherent to them. Adherence to Pap test recommendations declined from 87% to 83% between 1987 and 2005 [15]. Additionally, the percentage of individuals overdue for screening increased from 14% to 23% from 2005 to 2019 and worsened during the COVID-19 pandemic [6]. These findings demonstrate that declining screening participation was already occurring prior to the pandemic, with additional disruptions to preventive care further affecting screening adherence.

While some studies reported instances of over-screening, a greater proportion of patients were under-screened, with patterns strongly overlapping social determinants of health [6,15]. Data collected from 2013-2016 in a California healthcare system found that 18% of patients were over-screened, while 28% were under-screened [16]. Non-adherence was associated with obesity, low socioeconomic status, limited education, and lack of insurance coverage [16]. These findings demonstrate that screening adherence is influenced not only by screening recommendations, but also by socioeconomic and healthcare-access factors that may affect a patient's ability to obtain preventive care.

These same factors consistently predict lower screening participation across populations, with marginalized communities and developing countries at especially high risk due to limited access to preventive care. As recommended screening intervals become longer, patients who are already overdue or have difficulty accessing healthcare may experience even longer periods without screening. Expanding education, improving access to screening, and encouraging appropriate follow-up may therefore enhance adherence and reduce cervical cancer risk at a population level.

HPV Risk Factors/Epidemiologic Trends

Over 95% of cervical cancer cases are caused by HPV infection [1]. Regular screening could reduce cervical cancer mortality by approximately 70%; however, a lack of screening could increase the risk of cervical cancer fivefold [2]. Although many HPV infections naturally resolve, persistent HPV infection increases the risk of cervical cancer. Therefore, changes in HPV-related risk factors and epidemiologic trends remain important considerations when evaluating cervical cancer screening recommendations.

Having numerous sexual partners and a younger initial age of sexual intercourse increase the risk of HPV 16/18 infection by approximately 2.5-4 times [4]. Studies show that adolescents and adults in the USA have demonstrated trends involving a younger initial age of sexual intercourse, an increasing number of sexual partners, and a lack of consistent protective measures such as condom use [9,10]. These behaviors can increase opportunities for HPV exposure and transmission. Repeated exposures through new or multiple sexual partners may increase opportunities for HPV infection or reinfection. These sexual behavior trends are therefore important when considering HPV exposure and cervical cancer risk at a population level.

Nicotine use represents another concerning epidemiologic trend. Nicotine impairs HPV clearance by suppressing the immune response and allowing viral persistence, which may heighten the risk of cervical cancer. Studies also show increasing trends of nicotine and vaping use among youth, rising from approximately 11% to 28% between 2017 and 2019 [8]. Increased nicotine exposure among younger populations is therefore particularly relevant when considered alongside other HPV-related risk factors and the importance of clearing HPV infection.

Epidemiologic trends of increased sexual risk behaviors, reduced protective practices, increased nicotine use, and reduced screening adherence suggest an increase in HPV-related risk factors over time. With policy guidelines suggesting decreased screening frequency while epidemiologic risk factor trends indicate increased HPV-related risk, there may be a disconnect between policy recommendations and epidemiologic trends (Table 1). Current guidelines also generally recommend discontinuing screening after age 65 in appropriately screened individuals, yet approximately 20% of new cervical cancer cases between 2009 and 2018 occurred within this age group [17,18]. This demonstrates that cervical cancer continues to occur after the recommended age for screening cessation and suggests that late-age cervical cancer risk remains an important consideration.

**Table 1:** HPV risk factors vs. significance to cervical cancer vs. epidemiologic trends Epidemiologic trends demonstrate increased HPV-related risk factors associated with an increased risk of cervical cancer. HPV: human papillomavirus

HPV Risk Factor	Significance to Cervical Cancer	Epidemiologic Trend
Initial onset of sexual activity	Younger initial age of onset increases risk of early HPV exposure	Adolescents and adults in the USA from 1990-2018 have a decreasing initial age of sexual intercourse [4,9,10]
Number of sexual partners	Increased number of sexual partners increases risk of HPV exposure	Adolescents and adults in the USA from 1990-2018 have an increasing number of sexual partners [4,9,10]
Condom use	Decreased condom use increases risk of HPV exposure	Adolescents and adults in the USA from 1990-2018 have a lack of consistent protective measures such as condom use [9,10]
Screening adherence	Decreased adherence to screening increases risk of cervical cancer	Regular screening can reduce mortality by 70%; however, a lack of screening can increase risk of cervical cancer by five times [2]. Although screening guidelines end at 65 years of age, about 20% of new cervical cancer cases from 2009-2018 have been diagnosed for that age group [17,18]
Nicotine use	Nicotine impairs HPV clearance. Increased nicotine use increases risk of persistent HPV infection	Studies have shown that there are increased trends of nicotine and vaping use in youth rising from 11% to 28% when analyzing years 2017-2019 [8]

Overall, epidemiologic evidence demonstrates continued HPV-related risk factors occurring alongside guidelines that have shifted toward less frequent screening (Figure 1). Current HPV risk factor trends, declining screening adherence, and continued cervical cancer diagnoses among individuals over age 65 suggest a potential disconnect between current screening recommendations and epidemiologic trends. These factors should remain important considerations as cervical cancer screening recommendations continue to evolve.

**Figure 1:**
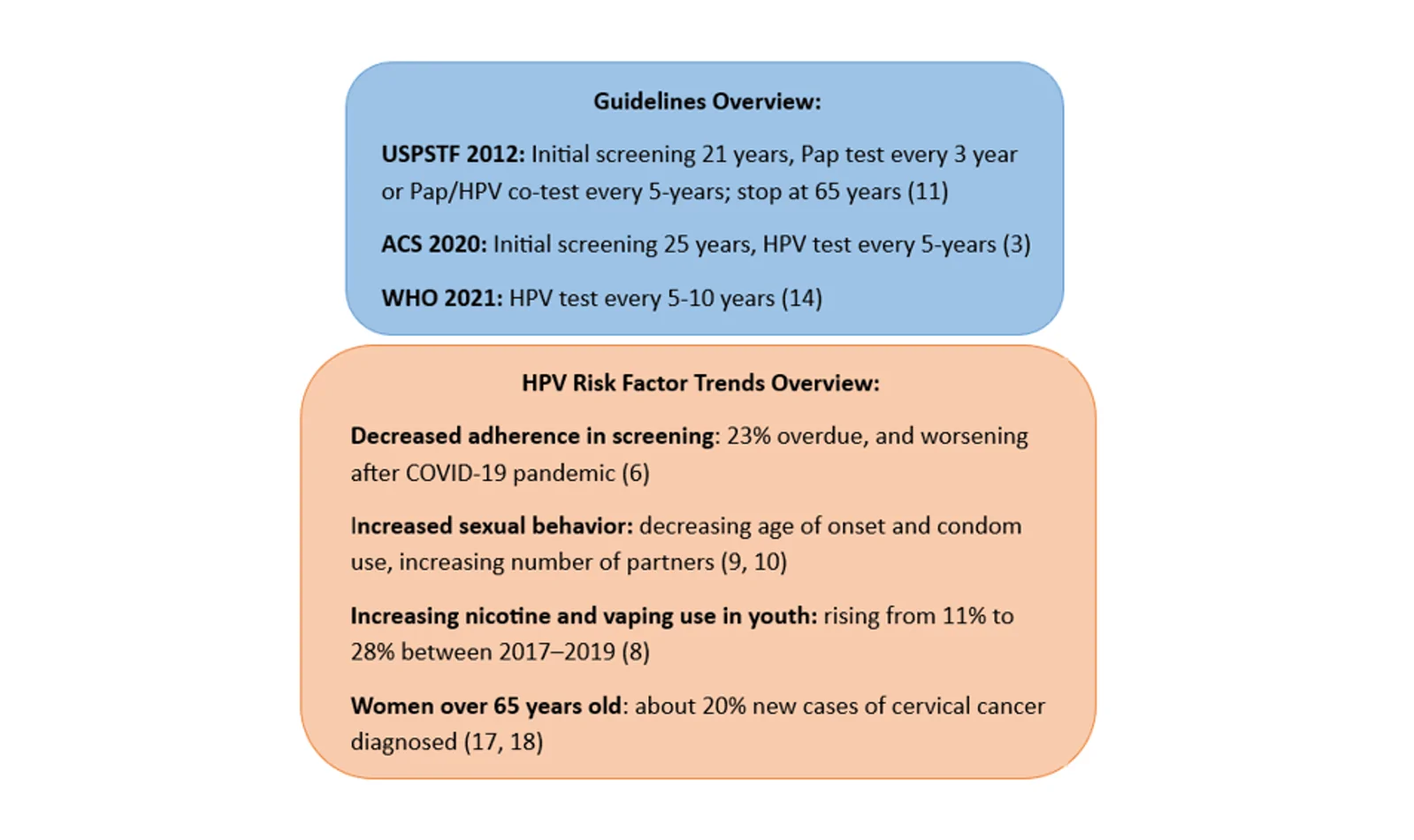
Cervical cancer screening guidelines vs. HPV risk factor trends overview Guidelines are trending toward decreased screening frequency, while HPV risk factor trends are increasing over time, suggesting a higher risk of HPV infection and therefore increased risk of cervical cancer. HPV: human papillomavirus

Communication, Comprehension & Provider/Patient Barriers

Patient-physician communication and comprehension strongly influence adherence to cervical cancer screening recommendations. Surveys from outpatient clinics across the USA in 2012-2013 revealed that 60% of patients were screened more frequently than recommended [19]. This pattern reflected residual habits following the 2012 guideline updates and suggests a delay in provider uptake of new recommendations. Some continued over-screening was attributed to provider preference for familiar practices, while other instances stemmed from patient expectations for annual testing [19]. These findings demonstrate that changes in screening guidelines may take time to be incorporated into routine clinical practice by both patients and providers.

When patients have greater access to information, understand screening guidelines, and receive direct recommendations from their physicians, they are more likely to remain within guideline-concordant screening intervals [20]. However, confusion regarding cervical cancer screening recommendations remains common. More recent surveys from 2021-2022 found that 27% of patients misunderstood current guidelines, believing annual Pap tests were still required, and 37% misreported the timing of their last screening [7]. These findings demonstrate that both understanding current recommendations and accurately recalling previous screening remain important factors in screening adherence.

Strong patient-physician communication could also reduce missed screenings and was associated with over a 1.6-fold higher adherence to cervical cancer screening recommendations [21]. Taken together, these findings highlight that misunderstanding, poor recall, and communication barriers may contribute to both over-screening and under-screening [7,19-21]. As screening intervals become longer, clear communication may become increasingly important to ensure that patients understand when they are due for screening and return at the recommended time. Clearer, ongoing communication between patients and providers may therefore improve adherence to screening recommendations and help reduce cervical cancer risk.

Strengths and limitations

This narrative review offers comprehensive information from sources addressing guideline changes, epidemiologic data, and HPV risk factor trends. This narrative review, however, lacks meta-analysis and does not include data on current trends and behavioral patterns following the COVID-19 pandemic, which limits the ability to quantitatively assess associations across studies and fully capture recent trends in epidemiologic risk factors. Despite these limitations, the narrative review approach serves as a strength by analyzing cervical cancer screening guideline policy recommendations in relation to epidemiologic risk factor trends, an important consideration for screening policy decisions.

Public health and policy implications

The policy guidelines and HPV risk factor epidemiologic trends are misaligned, with policy suggesting less frequent screening and epidemiologic trends suggesting increased risk of HPV infection and therefore cervical cancer. Guidelines are based on cost-effectiveness and the high sensitivity of HPV testing; however, they may not account for the reality of under-screening, non-adherence to recommendations, and behavioral trends that increase risk of HPV infection and cervical cancer at a population level. Given that non-adherence to screening overlapped with the social determinants of health, targeted interventions providing further education and access to marginalized communities and high-risk groups would benefit patients’ ability to adhere to the screening recommendations. Through this narrative review, recent cervical screening guideline changes were analyzed against the current HPV risk factor trends, an important aspect to consider when guiding future effective screening policies.

Future directions

Future policy guidelines should consider the epidemiologic trends of HPV risk factors displayed in this narrative review and consider if the increasing risk factor trends and decreased adherence rates are still outweighed by the cost-effectiveness of infrequent screening. Future data analysis can be conducted to view the most recent epidemiologic trends, especially after the COVID-19 pandemic, as initial data suggested adherence to screening decreased during the pandemic. Interventions to increase education and access to screening should be considered, especially in marginalized communities, as poor social determinants of health were associated with low adherence to screening. HPV self-sampling may be an interventional implementation to increase access to those who lack medical care, are uninsured, or have transportation or financial barriers to follow up with medical providers.

## Conclusions

This narrative review analyzed recent changes to cervical cancer screening guidelines in relation to current HPV risk factor trends. Over time, cervical cancer screening recommendations have shifted toward later initiation and less frequent screening based on advances in HPV testing, cost-effectiveness analyses, and increasing HPV vaccination. However, current epidemiologic trends demonstrate rising HPV-related risk factors, declining adherence to recommended screening, persistent patient-provider communication barriers, and continued cervical cancer diagnoses among individuals older than 65 years of age. Collectively, these findings suggest a potential disconnect between current screening recommendations and epidemiologic HPV risk factor trends. Continued evaluation of cervical cancer screening policies should consider evolving epidemiologic HPV risk factor trends alongside advances in screening technology to optimize cervical cancer screening strategies and further reduce cervical cancer incidence and mortality.
